# Chemical production of acidified activated carbon and its influences on soil fertility comparative to thermo-pyrolyzed biochar

**DOI:** 10.1038/s41598-020-57535-4

**Published:** 2020-01-17

**Authors:** Haider Sultan, Niaz Ahmed, Muhammad Mubashir, Subhan Danish

**Affiliations:** 1 Department of Soil Science, Faculty of Agricultural Sciences & Technology, Bahauddin Zakariya University, Multan, 60800 Punjab Pakistan; 2 College of Biology and Environment, Nanjing Forestry University, Nanjing, 210037 Jiangsu China; 3 Soil and Water Testing Laboratory, PakArab Fertilizer Limited, Khanewal Road, Multan, Punjab Pakistan; 4 Soil & Water Testing Laboratory for Research, Bahawalpur, 63100 Punjab Pakistan

**Keywords:** Plant sciences, Environmental impact

## Abstract

Biochar (BC) is gaining attention day by day due to its potential benefits for the improvement in degraded soil health. During its production by pyrolysis, carbon sequestration is an important aspect that makes it environment-friendly amendment. However, 100% anaerobic combustion of waste at such a high temperature decreases its adaptability to produce BC at commercial scale. On the other hand, the alkaline nature of BC also causes adverse effects on soil health when used in alkaline soils. Keeping in mind the problem of BC production and its high pH, current experiment was conducted to introduce chemical production of acidified activated carbon (AAC) and its effects on soil nutrients status comparative to high temperature pyrolyzed BC. As compared to thermal pyrolysis, sulphuric acid produce acidified activated carbon in minimum time and large in quantity. Sulphuric acid produces acidified activated carbon, fix higher carbon as compared to thermal pyrolyzed BC. Results also showed that application of 2% AAC was far better for decreasing alkaline soil pH*s* (3.52 and 4.71%) and EC*e* (45.2 and 71.4%) as compared to control in clay and sandy clay loam. A significant maximum increase in available P (117.5 and 25.9%), extractable Zn (42.0 and 52.2%), B (111.4 and 46.2%) and Fe (59.5 and 34.4%) in clay and sandy clay texture soils also validated the efficacious functioning of AAC over BC and control. It is concluded that sulphuric acid use is an easier and adaptable method to produce activated carbon at commercial scale. As compared to thermal pyrolyzed BC, application of AAC could be more effective in the improvement of soil health and fertility status.

## Introduction

Role of agriculture in food production is immense^1^ to fulfil the hunger of human’s^2^. Today modern agriculture is feeding more than 6000 millions of people, compared to old age hunter-gatherer lifestyle (that provide food to 4 million people)^3^. Owing to modern production technologies e.g. use of inorganic fertilizers, pesticides, organic amendments, biofertilizers and genetically modified high yield varieties, the production of crops has become double in the last 40 years^1,4,5^. This change in food demand and supply^6^ has shifted the conventional agriculture towards the intensive cultivation of crops^7^. However, intensive cultivation of crops not only depleted the concentration of the nutrients but also has degraded the soil health. Owing to high soil pH, low organic matter and poor microbial population, now crops are suffering from hidden hunger of macro and micronutrients^5,7,8^. To describe such a low concentration of macro and micronutrients bioavailability, poor soil fertility is a general term which is commonly used^9^. Since the early years of the 21^st^ century, the demand and application of inorganic fertilizers were increased tremendously to solve the problem of poor soil fertility^10^. It is documented fact that the application of inorganic fertilizers is the necessity of time to get the maximum yield of crops. However, these fertilizers are also costing a lot in terms of negative effects as well i.e., development of salinity problem^11^ in soil due to their enrich application^12^. Overuse of nitrogen and phosphorus fertilizers where increased crops productivity, they also have destroyed natural soil ecosystem^13,14^. Out of total applied nitrogen fertilizers, plants only consumed 50% while remaining 2–20% is lost due to its volatile nature as NH_4_, 15–25% chemically reacted with organic fractions and clay soil particle and 2–10% become part of water^15^. However, among micronutrients, the deficiency of Zn is another critical problem in alkaline calcareous soils which resulted in a significant reduction of yield^16–20^. An elevated level of P concentration in soil is also considered an important factor for the immobilization of Zn compared to high pH^21^. Enrichment of lakes with phosphorus due to its high rate of application is also causing eutrophication^22^. It has been observed that the deficiency of Fe also resulted in the chlorosis especially in citrus, deciduous fruits and leguminous crops^23,24^. The deficiency of boron has also played an imperative role in the deterioration of food quality^25–27^. Similarly, industries that are involved in the production of inorganic fertilizers are significantly contributing to heavy metals (Hg, As, Cd, Pb, Ni, and Cu) accumulation and contamination^28^. So far, many scientists have made attempts to resolve the problems of high soil pH and low organic matter by using organic amendments i.e., farmyard manure, compost and green manuring^29^. But their susceptibility towards decomposition is a major drawback. On the other hand, scientists also remained successful to tackle the problem of organic residues quick decomposition by introducing biochar (BC). Biochar is nutrients enrich environment-friendly organic amendment that is resistant against decomposition, decrease the emission of greenhouse gases (GHG’s) and sequester stable carbon^30^. Application of BC can significantly decrease the loss of nutrients by increasing soil CEC^31^. However, high pH of BC is again a major hurdle in the way to optimize the nutrients use efficiency, especially in high pH soils^32^. On the basis of BC pH, the buffering capacity of the soil is also increased towards change in soil pH^33^. The necessity of time is to make acidified BC. Although it is a very difficult task when produced at a commercial scale due to its high buffering ability. Keeping in mind the problems of low soil nutrients availability despite the presence of high immobile pool and high pH of biochar current experiment was conducted to introduce a chemical method for production acidified activated carbon (AAC). The aim of the current study was to introduce chemically carbon sequestration method for bulk production of AAC without using such a higher temperature. It is hypothesized that the use of the chemical method for production of AAC could be easier to adopt commercially, time-saving and less economic technique as compared to thermal pyrolysis.

## Results

### Soil pH_*s*_

**Figure 1:**
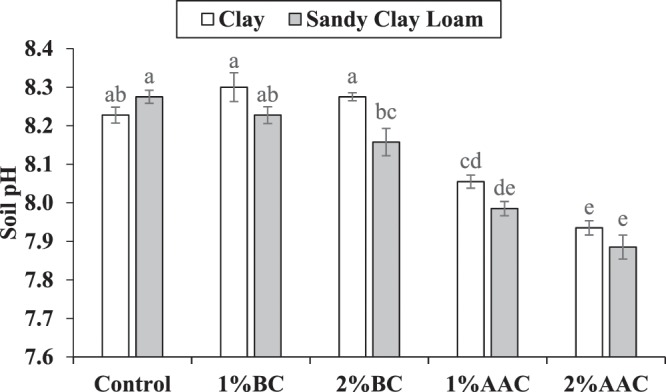
Effect of various levels of thermo pyrolyzed BC and chemically acidified activated carbon on clay and sandy clay loam textured soil pH*s*. Error bars represents standard error calculated through 3 replicates of each treatment.

Both main and interactive effects of various soil texture (ST) and treatments (T) were significant on the pH*s* of soil. No significant change was noted in soil pH*s* where 1% and 2% BC were applied as compared to control in clay. Addition of 1% BC was statistically alike in clay soil but 2% BC differed significantly in sandy clay loam for soil pH*s* as compared to control. It was observed that 1% and 2% AAC significantly decreased soil pH*s* over control in clay and sandy clay loam. Application of 2% AAC significantly decreased the soil pH*s* as compared to 1% AAC in clay soil (Fig. 1). However, 1% and 2% ACC remained statistically alike to each other in sandy clay loam soil. The maximum reduction of 3.52 and 4.71% in soil pHs was noted in 2% AAC over control in clay and sandy clay loam respectively.

### Soil EC_*e*_

**Figure 2:**
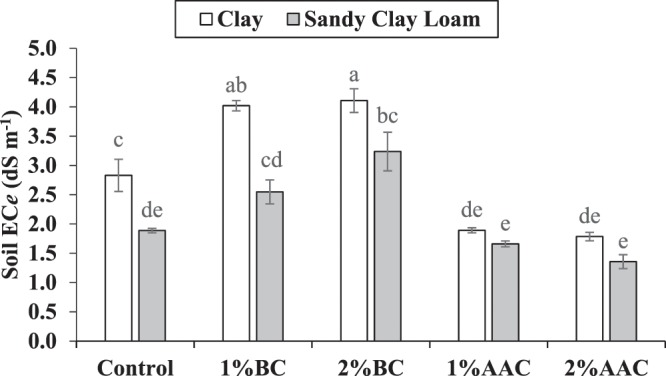
Effect of various levels of thermo pyrolyzed BC and chemically acidified activated carbon on clay and sandy clay loam textured soil EC*e*. Error bars represents standard error calculated through 3 replicates of each treatment.

Both main and interactive effects of various soil texture (ST) and treatments (T) were significant on EC*e* of soil. For soil EC*e*, the addition of 1% and 2% BC and AAC remained statically alike to each other but differed significantly as compared to control. Application of 1% and 2% BC significantly enhanced soil EC*e* over control in clay soil. However, 1% and 2% AAC significantly decreased soil EC*e* over control in clay soil. In sandy clay loam, no significant change in EC*e* was observed among control and 1% BC. However, 2% BC significantly increased EC*e* of sandy clay loam soil as compared to control. In addition, 1% and 2% AAC also remained statistically alike to each other and with control for soil EC*e* (Fig. 2). The maximum increase of 45.2 and 71.4% in soil EC*e* was noted where 2% BC was applied as compared to control in clay and sandy clay loam respectively. However, the application of 2% AAC gave the maximum reduction of 36.7% in soil EC*e* over control in clay soil.

### Soil phosphorus

**Figure 3:**
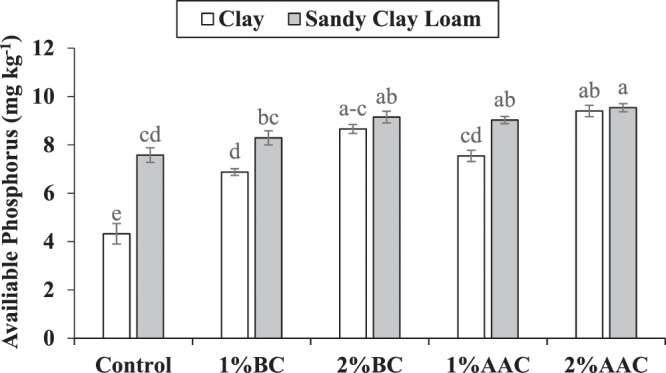
Effect of various levels of thermo pyrolyzed BC and chemically acidified activated carbon on clay and sandy clay loam textured soil available phosphorus. Error bars represents standard error calculated through 3 replicates of each treatment.

Both main and interactive effects of various soil texture (ST) and treatments (T) were significant on soil phosphorus (P). Application of 1% and 2% BC and AAC significantly improved the soil available P as compared to control in clay soil. For improvement in soil available P, 2% BC and AAC remained significantly better as compared to 1% BC and AAC in clay soil. No significant change in soil available P in clay soil was noted where 1% BC and 1% ACC were applied. Similarly, 2% BC and 2% AAC also remained statistically similar to each other for available soil P in clay soil. In case of sandy clay loam, 2% BC was significantly better as compared to control for available soil P. No significant change was noted among 1% BC and control for soil available P in sandy clay loam. However, 1% BC and 2% BC remained statistically alike to each other for available soil P in sandy clay loam (Fig. 3). It was observed that application of both 1% and 2% AAC performed significantly better as compared to control for available soil P. Application of 1% and 2% BC and AAC remained statistically alike to each other for available soil P in sandy clay loam. The maximum increase of 117.5 and 25.9% in soil available P was observed in clay and sandy clay loam respectively.

### Soil potassium

**Figure 4:**
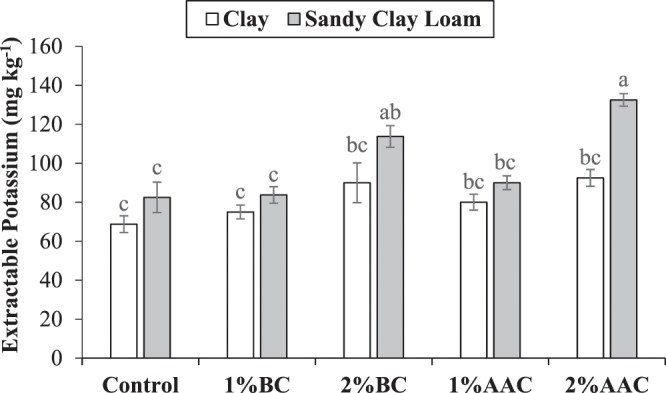
Effect of various levels of thermo pyrolyzed BC and chemically acidified activated carbon on clay and sandy clay loam textured soil extractable potassium. Error bars represents standard error calculated through 3 replicates of each treatment.

Both main and interactive effects of various soil texture (ST) and treatments (T) were significant on soil extractable potassium (K). No significant change was observed among 2% BC, 2% AAC, 1% BC, 1% AAC and control for extractable K in clay. In the case of sandy clay loam, application of 2% BC and 2% AAC remained significantly better over control for extractable K. Both 1% BC and 1% AAC were statistically alike to each other and with control for extractable K in sandy clay loam (Fig. 4). Similarly, 2% BC and 2% AAC also remained statistically alike to each other for extractable K in sandy clay loam. The maximum increase of 60.6% in extractable K was noted over control where 2% AAC was applied in sandy clay loam.

### Soil zinc

**Figure 5:**
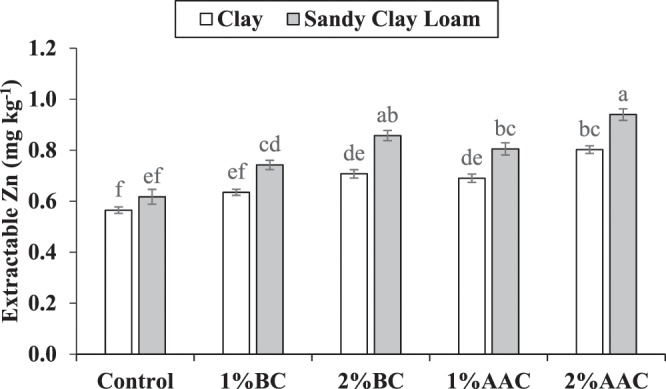
Effect of various levels of thermo pyrolyzed BC and chemically acidified activated carbon on clay and sandy clay loam textured soil extractable zinc. Error bars represents standard error calculated through 3 replicates of each treatment.

Both main and interactive effects of various soil texture (ST) and treatments (T) were significant on soil extractable zinc (Zn). Application of 2% AAC remained significantly better as compared to control for soil extractable Zn in clay. It was noted that 1% AAC, 1% BC and 2% BC were statistically alike to each other but only 1% AAC and 2% BC remained significantly better as compared to control for soil extractable Zn in clay. Application of 1% BC was statistically similar to control for soil extractable Zn in clay. In case o0f sandy clay loam, 2% BC and 2% AAC performed significantly better as compared to control for soil extractable Zn. No significant change was observed among 1% AAC and 1% BC for soil extractable Zn in sandy clay loam. It was observed that 2% BC was statistically alike but 2% AAC differed significantly as compared to 1% AAC for soil extractable Zn in sandy clay loam (Fig. 5). However, 1% AAC and 1% BC remained significantly better over control for soil extractable Zn in sandy clay loam. The maximum increase of 42.0 and 52.2% in soil extractable Zn in sandy clay loam was observed as compared to control where 2% AAC was applied in clay and sandy clay loam respectively.

### Soil boron

**Figure 6:**
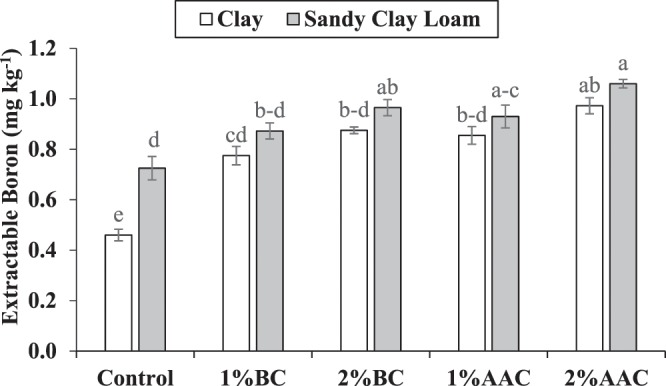
Effect of various levels of thermo pyrolyzed BC and chemically acidified activated carbon on clay and sandy clay loam textured soil extractable boron. Error bars represents standard error calculated through 3 replicates of each treatment.

Both main and interactive effects of various soil texture (ST) and treatments (T) were significant on soil extractable B. Application of 2% BC, 1% AAC and 2% AAC were statistically alike to each other but differed significantly as compared to control for soil extractable B in clay. No significant change was noted among 1% BC, 2% BC and 1% AAC for soil extractable B in clay. However, 2% BC remained significantly better as compared to 1% BC for soil extractable B in clay. In addition, 1% BC also differed significantly for soil extractable B over control in clay soil. In the case of sandy clay loam, application of 1% BC, 2% BC, 1% AAC and 2% AAC were statistically similar to each other from control for soil extractable B (Fig. 6). However, 2% BC, 1% AAC and 2% AAC differed significantly from control for soil extractable B in sandy clay loam. No significant change was noted in soil extractable B over control in 1% BC. The maximum increase of 111.4 and 46.2% in soil extractable B was noted in 2% AAC over control in clay and sandy clay loam respectively.

### Soil iron

**Figure 7:**
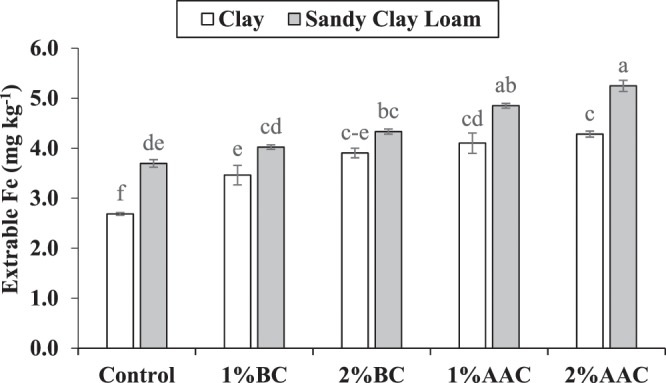
Effect of various levels of thermo pyrolyzed BC and chemically acidified activated carbon on clay and sandy clay loam textured soil extractable iron. Error bars represents standard error calculated through 3 replicates of each treatment.

Both main and interactive effects of various soil texture (ST) and treatments (T) were significant on soil extractable iron (Fe). Application of 2% BC, 1% AAC and 2% AAC were statistically alike to each other but differed significantly as compared to control for soil extractable Fe in clay. No significant change was noted among 2% BC and 1% BC for soil extractable Fe in clay soil. It was noted that 1% BC also remained significantly different as compared to control in clay for soil extractable Fe. For sandy clay loam, 1% and 2% AAC were statistically similar to each other but differed significantly as compared to control for extractable Fe (Fig. 7). Similarly, no significant change was noted among 1% BC and 2% BC but only 2% BC remained significantly better from control for extractable Fe in sandy clay loam. The maximum increase of 59.5 and 34.4% in extractable soil Fe was noted over control where 2% AAC was applied as a treatment in clay and sandy clay loam respectively.

## Discussion

In the current study, the application of BC significantly increased the pH*s* of soil. This increase in soil pH*s* was due to high pH of BC. Presence of alkaline mineral elements was the possible reason for the high pH of BC (Table 1). Biochar addition in soil significantly enhanced the soil EC*e* possibility due to the release of mineral nutrients (soluble base cations K^+^ and Ca^++^ and Na^+^) in soil solution and exchange with soil exchange sites. Pre-experimental analysis of P, K and Ca in BC (Table 1) validated our argument regarding a significant increase in soil EC. A significant amount of presence of mineral nutrients in BC structure is well documented by many scientists^5,34,35^. Abid *et al*.^34^ also reported similar kind of significant increase in soil pH when they applied BC in soil under various sources of irrigation. During pyrolysis oxygen-containing functional groups and alkaline ash, contents are increased on the surface of BC that played an imperative role in the enhancement of soil pH^36^. According to Yuan *et al*. ash content of BC may have 48–330 cmol kg^−1^ soluble base cations (K^+^, Ca^++^, Mg^++^, and Na^+^). On the other hand, the presence of Ca^++^, Mg^++^ and K^+^ in large amount easily replace the H^+^ ions in soil solution, thus, resulted in an increase of soil pH^37^. However, AAC significantly decreased the soil pH*s* might be due to the presence of H_2_SO_4_ in the pore spaces. The molecules of H_2_SO_4_ possibility become the part of soil solution when the soil was irrigated. Enrichment of H^+^ ions by H_2_SO_4_ decreased the soil pH significantly. Furthermore, higher amount of Ca in calcareous soil might react with SO_4_ which make CaSO_4_ that is well documented regarding aaleviation of adverse effects of Na. In addition, AAC would be the major cause of an increase in the buffering ability of soil to sustain low pH for a long time (60 days). Reduction in soil EC by AAC might also be due to sorption of minerals by empty exchange sites, possibly produced due to the release of H_2_SO_4_ in soil. Low pH of soil played an imperative role in the solubilization of immobile soil P. According to Hopkins and Ellsworth^38^ reduction in soil pH (6.5) increase the mobility of fixed soil P. Under low soil pH, the activity of H^+^ dissociates the linkage of calcium and phosphorus in calcareous parent material soils. The breakage of the bond between calcium and P resulted in dissolution and availability of immobile P in soil solution^39^. In addition, P is also the part of BC structure as well^40^. Reduction in pH of biochar also increases the release of P from biochar into soil solution^41^ as observed in AAC of the current study. Indirectly BC application also facilitates the soil microbes to secrete phosphomonoesterase that enhances the soil P mineralization^42^. In the current study, improvement in micronutrients, Zn, B and Fe might also be associated with the reduction in soil pH and improvement in cation exchange sites of soil. According to Laird^43^ application of BC can increase 20% cation exchange capacity (CEC) of soil. This CEC is an indirect measure which enhanced water and nutrients retention by decreasing its leaching loss. Higher surface area and the carboxyl group of BC play an imperative role in increasing the CEC of soil and nutrients availability^44^.

**Table 1:** Characteristics of thermo pyrolyzed biochar (BC) and acidified activated carbon (AAC).

BC	Unit	Value	AAC	Unit	Value
pH	—	4.11	pH	—	8.03
EC_*e*_	dS m^−1^	1.83	EC_*e*_	dS m^−1^	2.11
Volatile Matter	%	10.90	Volatile Matter	%	12.29
Ash Content	%	13.10	Ash Content	%	40.26
Fixed Carbon	%	76.00	Fixed Carbon	%	47.45
Total N	%	0.95	Total N	%	0.16
Total P	%	0.66	Total P	%	0.43
Total K	%	2.09	Total K	%	1.66
Total Ca	%	4.21	Total Ca	%	3.96
Total Na	%	0.61	Total Na	%	0.59
Total Zn	mg kg^−1^	0.10	Total Zn	mg kg^−1^	0.13
Total B	mg kg^−1^	0.17	Total B	mg kg^−1^	0.22
Total Fe	mg kg^−1^	1.12	Total Fe	mg kg^−1^	1.01

## Conclusion

In conclusion, sulphuric acid use is an easier and adaptable method to produce activated carbon at commercial scale. Thermal pyrolyzed BC due to high pH is less efficacious than AAC for improvement in soil health and fertility status. More investigations are needed to introduce AAC as an effective replacement of BC optimum utilization of micro and macronutrients in soil.

## Materials and Methods

An incubation experiment was conducted in Soil and Water Testing Laboratory for Research Multan. The treatments were control (no BC and no AC), 1% biochar (1% BC), 2% biochar (2% BC), 1% acidic activated carbon (1% AAC), 2% acidic activated carbon (2% AAC) applied in two different texture of soil (clay and sandy clay loam).

For the production of thermo pyrolyzed biochar (BC), sugarcane waste syrup was collected from the sugar mill. After oven drying at 65 °C, clods like the structure of syrup was collected and pyrolyzed in partially aerobic pyrolyzer at 550 °C for 75 min^5^. Finally, prepared BC was grinded and passed through sieve 2 mm sieve and stored in airtight plastic jars for further experimentation.



For the very first chemically acidified activated carbon (AAC) was prepared by using sugarcane waste syrup of sugar mill. Syrup waste was taken in a specially designed reactor. After that concentrated (98%) sulphuric acid (H_2_SO_4_) was added in the reactor (2:1, v/v). A vigorous reaction takes place in which water was evaporated from the waste syrup of sugar mill leaving behind acidic activated carbon (AAC).

$${\rm{Carbohydrates}}({{\rm{CH}}}_{2}{\rm{O}})+{{\rm{H}}}_{2}{{\rm{SO}}}_{4}({\rm{acidifying}}\,{\rm{and}}\,{\rm{dehydrating}}\,{\rm{agent}})={\rm{AAC}}+{{\rm{H}}}_{2}{\rm{O}}$$

Biochar pH and EC were determined in BC and AAC by making water ratio of 1:20 w/v^32^. Digestion of BC and AAC was done by using di-acid mixture HNO_3_:HClO_4_ in 2:1 ratio^45^. The yellow colour method was followed for total phosphorus (P) analysis in BC and AAC on a spectrophotometer^46^. Potassium concentration in BC and AAC was measured on flamephotometer^47^. For analysis of nitrogen on Kjeldahl’s distillation apparatus^48^, H_2_SO_4_ digestion^46^ was followed. Ash content (AC) and volatile matter (VM) in BC and AAC were determined by heating the sample in a muffle furnace at 550 °C and 450 °C respectively^49^. The fixed carbon in BC and AAC was calculated using the equation Noor *et al*.^50^:

$${\rm{Fixed}}\,{\rm{Carbon}}( \% )=100-( \% {\rm{Volatile}}\,{\rm{Matter}}+ \% {\rm{Ash}}\,{\rm{Content}})$$

The characteristics of BC and AAC are given in Table 1.

For incubation of soil, small clay pots were used. In each pot, 1 kg of soil was added along with BC and AAC as per treatment plan. The moisture of soil was maintained 65% on w/w basis throughout the incubation of 60 days. After 60 days all the soil samples were initially air dried and then passed through 2 mm nylon sieve for their analysis.

For determination of pH_*s*_ and EC_*e*_ of soil Schofield and Taylor^51^ and US Salinity Laboratory Staff ^52^ were followed respectively. Extractable soil phosphorus was analyzed by Olsen and Sommers^53^ methodology. Nadeem *et al*.^47^ method was followed for determination of extractable soil K. Boron in soil samples were analyzed according to Bingham^54^ on a spectrophotometer using Azomethine-H. Micronutrients zinc (Zn) and iron (Fe) were analyzed on atomic absorption spectrophotometer according to the methodology of Lindsay and Norvell^55^.

Statistical analysis was done according to the standard statistical procedure of Steel *et al*.^56^. Descriptive statistical analysis and analysis of variance (ANOVA) was applied on data to find significance. Means were compared by Tukey’s test and correlation was find at the *p* ≤ 0.05 level^47^.

## Data Availability

No datasets were generated or analyzed during the current study. All the analyzed data can be accessed after publication by requesting to the corresponding author.
